# Pseudo Tuberculosis

**Published:** 1891-04

**Authors:** A. W. Clement


### PSEUDO-TUBERCULOSIS.

BY DR. A. W. CLEMENT.

Through the kindness of Dr. Ward, State Veterinarian, I had opportunity of making an autopsy, the record of which I herewith present.

Subject.—A Ewe, six years old, killed by bleeding for purpose of autopsy.

EXTERNAL APPEARANCES.—Body somewhat emaciated. Mucus membranes pale; otherwise normal. Skin normal. *Subcutaneous.*—Very little adipose tissue. Lymphatic glands normal. Mammæ normal.

ABDOMEN.—Muscle pale. No fluid in cavity. Surface of peritoneum smooth and glistening. No adhesions. Between the layers of the mesentery are several hard, circumscribed nodules, some of them the size of a pea; others five or six times as large. These nodules are scattered irregularly between the folds. On section they present a caseous centre surrounded by a fibrous capsule. The caseous material is mixed with calcareous particles. Beneath the visceral layer of peritoneum, especially beneath the portion covering the coil of large intestine are to be seen a large number of hard nodules, some circumscribed and the size of a pea, others confluent covering an area about the size of a silver quarter dollar. The surface of the nodules is rough and uneven. These nodules, on section, show a caseous centre mixed with calcareous particles, and a fibrous periphery; also none of the nodules examined are any entozoa to be seen. In the fold of the omentum is a nodule, dark red in color, smooth on surface, and not hard. On section, this nodule is seen to be a blood clot with a grayish central portion, about the size of a pin's head. There does not appear to be anything comparable to a wall surrounding this area. Close to this area, however, is a second nodule having the same external appearance but somewhat smaller than the first. On section there is a grayish central portion about the size of a pin's head, surrounded by a dark red area which is distinctly adherent to a fibrous wall. The inner surface of this wall is roughened where the contained clot is pulled off. This nodule ends abruptly but there appears to be a small vessel leading from it. The grayish, central portion, when examined under the microscope, is seen to be made up of fibrin fibrils. *Spleen.*—Surface smooth and glistening. The organ is normal in size and of normal, firm consistency. On section, bright red in color; pulp firm. *Pancreas.*—Surface smooth and glistening. On section, normal in consistency and color. *Liver.*—Surface smooth and glistening except at lower border of right lobe where just beneath Glisson's capsule is a nodule the size of a hickory nut. The surface of this nodule is rough and uneven. On section, it is calcareous throughout. The substance of the organ is firm and of normal brown color. The gall bladder is partly filled with dark-green bile. Portal veins empty. *Kidneys.*—Capsule easily detatched, leaving surface of organ smooth. On section, this appeared normal. *Suprarenal Capsules.*—Normal on surface and on section. *Uterus* normal. *Bladder* empty. Mucus membrane smooth. Beneath the mucus membrane there are a considerable number of small, striated hemorrhages.

THORAX.—No fluid in cavity. No adhesion. *Pleural* surface smooth and glistening, so far as can be seen with the organs in situ. *Pericardium.*—About a tablespoonful of clear, straw-colored fluid in sack. Surface smooth and glistening. No adhesions. *Epicardium* smooth and glistening. No hemorrhages beneath this membrane. *Heart muscle* normal. Chambers empty. *Endocardium* normal. No sub-endocardiac hemorrhages. *Lungs.*—Crepitant throughout. Pleural surface smooth and glistening. Just beneath the pleura, more especially on the superior border of the lung are a great number of pin-head sized, semi-opaque bodies with a steel-gray periphery and a grayish-white, more opaque central portion. These bodies do not shell out. *Lymphatic glands* in medio-stinum normal. *Bronchial mucous membrane* smooth; and normal in color. No mucus in tubes and no strongylus.

THROAT AND MOUTH.—Organs normal.

BRAIN.—Dura mater adherent to skull cap. Pia mater smooth. Blood vessels not prominent. No fluid in ventricles; and glistening. Choroid plexus normal. Arteries at base of brain normal. Substance of brain normal.

SPINAL CORD not examined. The *tubercle* in lungs show, in traced preparation under the microscope; a small nematode worm corresponding to the grayish-white central portion. Further study of the entozoa and their possible or probable connection with the tubercle in the abdominal cavity deferred.

MARCH 5, 1891.

Upon further examination I find that the tubercles in the lungs were caused by the "Strongylus ovis pulmonalis." A good description of this parasite will be found in the "Animal parasites of sheep."—Bureau of Animal Industry, Washington, 1890.

